# Maintenance in relationships, satisfaction, jealousy, and violence in young couples: a network analysis

**DOI:** 10.1186/s40359-023-01411-z

**Published:** 2023-11-09

**Authors:** José Ventura-León, Cristopher Lino-Cruz, Tomas Caycho-Rodríguez, Christian Córdova-Robles

**Affiliations:** 1 Universidad Privada del Norte, Lima, Perú; 2 Universidad Peruana de Ciencias Aplicadas, Lima, Perú; 3 Universidad Científica del Sur, Lima, Perú

**Keywords:** Relationship maintenance, Satisfaction, Jealousy, Violence, Young couples, Network analysis, Pandemic

## Abstract

**Background:**

The study explores the associations among Relationship Maintenance, Satisfaction, Jealousy, and Violence in young Peruvian couples, particularly in a post-pandemic context, using a network analysis.

**Methods:**

Eight hundred thirty-two participants aged 18–30 (M = 20.94, SD = 2.29), with 645 females (77.50%) and 187 males (22.50%), were involved. The study aimed to discern relationships among network nodes, emphasizing the link between Relationship Maintenance dimensions and Jealousy and Violence. The research also sought the central node in the network and examined gender-specific node connections, using the SMOTE algorithm for gender data balance.

**Results:**

Findings revealed a direct connection between Complementarity and Jealousy, implying intense shared interests can lead to unhealthy dependence. An inverse relationship was seen between Companionship and Violence. Satisfaction was pivotal, showcasing its importance in romantic relationship success. Additionally, the study shows men prioritize Companionship and Sharing, possibly due to cultural norms, while women focus on the Companionship-Complementarity bond, indicating mutual support.

**Conclusions:**

The research emphasizes the critical role of maintenance variables in determining Satisfaction, Jealousy, and Violence in relationships. The pandemic's influence on romantic dynamics is evident, emphasizing the importance of Satisfaction. Future studies should focus on gender equity and further explore these relationships.

**Supplementary Information:**

The online version contains supplementary material available at 10.1186/s40359-023-01411-z.

## Introduction

The National Institute of Statistics and Informatics in Peru has documented a concerning trend: the population of separated and divorced individuals has increased while the population of married individuals has dwindled between 1993 and 2017 [1]. This shift has profound implications for the understanding of romantic relationships in the Peruvian context, especially given the significance young individuals attribute to these relationships as a contributing element to personal happiness [2]. Given these developments, the domain of romantic relationships in Peru merits an in-depth exploration, particularly focusing on factors like marital satisfaction, integration, stability, and relational maintenance behaviors [3]. Global relationship dynamics have shifted notably. Over recent decades, countries like Canada and the U.S. have seen rising divorce rates [4]. This shift, often attributed to societal changes [5] and economic strains [6], has ignited scholarly debates. Post-COVID-19, nations such as Spain and Italy have also observed declining marriage rates [7]. The challenges posed by the pandemic, including imposed isolations and socio-economic uncertainties, have intensified relational strains globally [8, 9]. While these global trends provide a backdrop, it's crucial to understand how they intersect with or diverge from the unique socio-cultural dynamics in Peru. By juxtaposing these global trends with the Peruvian context, we can derive insights into the distinctive factors shaping romantic relationships in the region and inform localized interventions and support mechanisms. This comparative approach enriches our investigation, allowing us to contextualize our findings within a global narrative. Delving into the intricate phenomenon of romantic relationships, particularly in the Peruvian context, necessitates a comprehensive examination of the variables involved. This article will provide an introduction, delve into relationship maintenance behaviors and factors affecting relationships such as jealousy and violence in Peru, explore the methodological approach, discuss the clinical relevance, and conclude with the study objectives.

Maintenance behaviors have been defined as the activities that couples engage in to preserve and prevent a decline in their romantic relationships [10, 11]. Previous research has shown variations in the types and effectiveness of these behaviors in different cultural contexts, finding that these behaviors are more common in females, and it has been found that the attachment variable may help explain these differences [12]. In fact, five behaviors have been initially identified as the basis for successful romantic relationships: positivity, openness, assurance, social networks and shared tasks [13]. In this scenario, maintenance behaviors enhance the quality of interaction and promote healthy bonds between couples [14]. Therefore, they are indicators of relational stability that help prevent a decline or potential breakup of the romantic relationship [15, 16]. This requires the presence of elements such as mutual commitment, effective communication, complementarity in the relationship, and expressions of affection and companionship [17, 18]. Indeed, companionship, humor, task collaboration, and verbal expression of affection have been identified as the most valued aspects by adult couples in their romantic relationships [18]. In general terms, companionship is essential in romantic relationships as it entails the desire for long-term commitment and the pursuit of mutually satisfying interactions [19]. Therefore, maintenance behaviors are critical indicators for satisfaction in romantic relationships, as demonstrated in previous research studies [20]. Relationship satisfaction refers to the subjective evaluation that a person makes of his or her love relationship in the present [21], which is considered to be a key predictor of success and durability in romantic relationships [22]. In this sense, both satisfaction and love are essential elements in romantic relationships [23]. This fact becomes relevant as, in some countries, both dating and married couples experienced a significant decrease in their levels of satisfaction and love after the end of the COVID-19 emergency [24]. Such shifts underscore the urgent need to investigate how relationship dynamics, including maintenance behaviors and challenges like jealousy and violence, have evolved in this new context. Relationship satisfaction has been related to maintenance behaviors [14], with the quality of the relationship and satisfaction with life [25]. However, evidence of a negative relationship with jealousy has been found [26].

Jealousy is considered an inherent emotion in love relationships, which arises from a real or imagined suspicion of a threat of loss of affection from a relationship considered to be of great value [27]. Other studies have also pointed to factors such as insecurity and past relational traumas as contributors to jealousy [28]. Thus, it has been suggested that jealousy and distrust can be seen as a form of negative relationship maintenance [29, 30]. It has been shown that expressions of jealousy vary according to gender, as women tend to express jealousy accompanied by feelings of sadness or depression, while men tend to express it through anger or aggression [31]. This is related to a systematic review study conducted by Pichon [32], who showed that distrust and jealousy were strongly associated with intimate partner violence.

Violence in the relationship refers to the attempt to exert dominance and control over the other person, either physically, psychologically or sexually [33]. Previous research has underscored the multifaceted nature of relationship violence, linking it to factors such as power dynamics [34] and societal norms and behaviors to which some men resort to justify their use of gender-based violence [35]. In fact, the presence of violence in romantic relationships is a predictor of low levels of satisfaction, trust and closeness between couples, hindering the full development of the couple as it obstructs the fulfillment of both partner and personal needs [36].

While prior studies have explored the individual dynamics of relationship maintenance, satisfaction, jealousy, and violence, few have integrated these aspects into a comprehensive network analysis. The lack of research that holistically assesses the interplay of these variables presents a gap in our understanding. This study, therefore, seeks to fill this void by employing a network analysis approach. To comprehensively explore the dynamics at play, we delve into multifaceted aspects of romantic relationships, such as complementarity, directly linked to satisfaction, relationship erosion, and physiological functioning post-conflict [37]. Affectivity significantly influences relationship satisfaction and romantic love, particularly in terms of intimacy, encompassing support provision, reception, and effective communication [38, 39]. Companionship assumes a fundamental role, with shared novel activities enhancing relationship satisfaction [40]. Married women highlight companionship's value, offering presence, support, care, and trust for shared experiences and conversations [41]. Allocating quality time together emerges as pivotal, benefiting the relationship's quality [42] and individual enjoyment, promoting happiness during shared activities [43]. Understanding the dynamics necessitates acknowledging differing expectations and perceptions between genders [44]. Notably, women tend to exhibit higher dissatisfaction and contemplate separation, leading to increased divorce initiation rates [45]. Complex factors contribute to such dissatisfaction, including labor division inequalities, varied expectations, and divergent notions of fairness and justice [46, 47].

Considering the unprecedented relational challenges posed by the post-COVID-19 era, there's a compelling need for advanced analytical methods to understand these complexities. On this note, while traditional scientific evidence indicates that correlational studies between jealousy and satisfaction, as well as aggression and jealousy, have been conducted using the Pearson correlation [26, 48], newer methodologies like network analysis offer deeper insights. However, no studies have been found that correlate variables using network analysis, which is a method considered novel and potentially more efficient than latent variable modeling for studying psychological attributes [49, 50]. Network analysis has been increasingly applied as a novel approach to understanding the nature and treatment of various variables associated with mental health in different domains [51]. In network analysis, symptoms of mental health are nodes that interact and mutually reinforce each other within a network [49]. To achieve this, network analysis allows for the representation of relationships within and between mental health variables [49]. Although network analysis in psychology was initially used to analyze psychopathological variables, there is no doubt that in recent years it has been expanding to other areas of psychology, such as intelligence, psychology and psychology [52], personality [53], emotional intelligence [54], academic self-efficacy [55], and even in the field of love relationships [56]. Network analysis assesses the strength and nature of associations between nodes, disregarding the assumption that the summation of scores on these variables describes psychological characteristics [57]. In this way, network analysis allows for the identification of central nodes, which are those with stronger connections to other nodes [58]. Previous studies using network analysis in other fields have underscored its ability to provide nuanced insights into complex systems, showcasing its potential utility in the realm of romantic relationships [56, 59]. Thus, studying the relationships between relationship maintenance, satisfaction, jealousy, and violence in young couples using network analysis has clinical utility, as it enables an understanding of variable-to- variable interactions [60]. Furthermore, it is potentially useful for identifying interventions in working with couples that may be effective in treating individual syndromes [61].

Therefore, the present study aims to estimate the network structure of nodes between relationship maintenance, satisfaction, jealousy, and violence in young couples in Lima Metropolitana. Additionally, it seeks to identify the interconnections between nodes, the central node, and compare the network based on gender.

## Method

### Participants

The participants were 832 young people and adults aged between 18 and 30 years (Mean = 20.94, *SD* = 2.29); 645 females (77.50%) and 187 males (22.50%). Each and every participant was engaged in a romantic liaison lasting a minimum duration of three months, as it was regarded imperative for achieving a requisite level of stability [62]. In general, the duration of the romantic partnership varied from 3 to 139 months (Mean = 22.56, *SD* = 19.97). All participants were from a middle socioeconomic stratum and belonged to the city of Metropolitan Lima. The sample size was predetermined by utilizing the *powerly* package, with 10 nodes, a statistical power of 0.80, and a density of 0.40, which indicated that a minimum of 262 observations was recommended [63]. The process of participant selection was accomplished by utilizing a non-probability technique known as snowball sampling [64]. This was due to the impact of the COVID-19 pandemic on the traditional in-person and large-scale surveying practices in Peru.

### Instruments

All the instruments used in this section are adaptations of other authors that have been appropriately validated in the Peruvian context, which guarantees their use. In the case of the WAST-2, its psychometric properties have been examined as a preliminary aspect for its use (see Supplementary Information).

### The Relationship Maintenance Scale (RMS) [65]

A 14-item Peruvian version of the RMS was used [66]. The RMS consists of Likert-type items ranging from 1 (Strongly Disagree) to 5 (Strongly Agree). The RMS measures four factors: Companionship, Affection, Complementarity, and Shared interaction. For example, some of the items indicate We share ideals, We feel chemistry in our relationship, We talk about what happens to us. The validity testing was conducted through exploratory and confirmatory factor analysis using the WLSMV estimator, and the goodness-of-fit indices were optimal (CFI = 0.977, RMSEA = 0.058) according to previous studies [67]. Reliability was assessed using the omega coefficient, which showed acceptable to good internal consistency measures for Companionship (ω = 0.78), Affection (ω = 0.83), Complementarity (ω = 0.77), and Sharing (ω = 0.70). Values that may be relevant [68, 69].

### Relationship Assessment Scale (RAS) [21]

A five-item Peruvian version of the RAS was used [70]. It comprises a Likert-type scale ranging from 1 to 5. This unidimensional questionnaire measures the level of satisfaction in romantic relationships. The RAS-5 is in the range of 5 to 25 points. For example, some of the items indicate Do you feel that your partner meets your needs? Overall- to what extent are you satisfied with your relationship, how good is your relationship compared to most couples? Two approaches were used to evaluate the validity of the questionnaire: Item Response Theory (IRT) and Confirmatory Factor Analysis (CFA). Both approaches demonstrated excellent goodness-of-fit, with an RMSEA below 0.08 and a CFI above 0.95. Furthermore, reliability was assessed using two coefficients: empirical reliability (*r*_*xx*_ = 0.86) and omega coefficient (ω = 0.84), indicating a high level of internal consistency [69].

### The Woman Abuse Screening Tool (WAST-2) [71]

The Spanish version was used [72]. This instrument consists of two Likert-type items. The WAST-2 has a score in the range of 0 to 4. The instrument is unidimensional and designed to assess the presence of violent outbursts, tension, and difficulties in romantic relationships. The scale reliability was considered acceptable [68] for the study sample (ω = 0.66). While the WAST-2 was originally designed as an instrument to assess violence against women, its two items (1. Overall, how would you describe your relationship with your partner? □High tension, □Some tension, □No tension; 2. You and your partner resolve disagreements with: □A lot of difficulty, □Some difficulty, □No difficulty) are broad enough to measure episodes of violence in both genders.

### The Brief Jealousy Scale (BJS) [73]

The BJS is a scale from one of the dimensions of the Inventory of Emotional Communication in Romantic Relationships [74]. The BJS consists of nine items rated on a Likert scale ranging from 1 (Not jealous at all) to 5 (Very jealous), assessing various scenarios in which an individual may experience jealousy. For example, some items indicate If my partner spends much more time with another person, I would feel or If I feel that my partner trusts another person more than I do, I would feel. The validity of the scale was established through confirmatory factor analysis, demonstrating an acceptable goodness-of-fit (CFI = 0.97; SRMR = 0.03; RMSEA = 0.08) according to previous studies [67]. Additionally, reliability was determined using the omega coefficient (ω = 0.88) which can be an indicator of good internal consistency [68].

### Procedures

Before initiating the research, an evaluation of ethical considerations stipulated in the Helsinki Declaration [75] and aspects related to conducting online research was conducted [76]. This was presented to the Research Ethics Committee of Universidad Privada del Norte (UPN) in Peru. Initially, the WAST-2 was analyzed, the only instrument without a psychometric study in the Peruvian context. Given the test's brevity, Item Response Theory (IRT) models were employed to assess differential functioning by gender. Specifically, the Expected Score Standardized Difference (ESSD) was used, which is based on expected scores and provides a measure of the effect size in the latent trait [77]. A value of ESSD > 0.30 indicates a small effect, ESSD > 0.50 is moderate, and ESSD > 0.80 is large. The results were favorable, allowing for the inclusion of the WAST-2 (see Supplementary Information).

Due to the limitations and difficulties that arose following the COVID-19 pandemic in accessing participants through traditional means, a non-probabilistic snowball sampling method was chosen. While this approach might have its downsides, like potentially skewing towards specific societal segments, it was crucial for gathering information in the challenging post-pandemic landscape. Opting for this sampling technique mainly stemmed from our aim to gather as many participants as possible, while also tapping into the interconnected web of personal relationships and social circles. As such, participants were invited to participate through initial contacts who, in turn, recommended other potential participants. While we acknowledge that this method may limit the generalizability of our results, it was a pragmatic solution given the post-pandemic circumstances.

Once contact was established with potential participants, they were provided with a consent form that detailed the study's objectives, anonymity assurance, potential risks and benefits, and data handling protocols. Subsequently, they were administered a sociodemographic questionnaire, which helped contextualize responses and understand the diversity of the sample. After completing this preliminary questionnaire, they proceeded to respond to self-reported questionnaires about their romantic relationships. These questionnaires included specific questions designed to capture the dynamics of romantic relationships. Participants were encouraged to answer with honesty, and we emphasized that their responses would be met without any form of judgment or consequence. On average, it took about 15 min for individuals to complete the entire questionnaire suite, with the data gathering period spanning from March to June 2022. Comprehensive details of the data and R code were archived in the free OSF repository: https://osf.io/vbyhq/.

### Data analysis

The R programming language was used to perform data analysis within the RStudio environment. The protocol recommended by the reporting standards for psychological network analyses was followed [78]. As a result, the network estimation, accuracy assessment, stability, and comparative analysis were conducted.

Prior to the network analysis, an exploration of the variables or nodes of interest was conducted using Global Network Properties to describe the network. This included the density (D), which represents the proportion of existing connections in the graph; the transitivity (C△), which measures the average tendency of nodes to form groups or communities in the network; and the average shortest path length (APL), which indicates the average number of links or connections required to reach from one node to another in the network. Lastly, the small-world index (S) was calculated, which evaluates the degree of association between nodes, with a recommended value greater than 1 [79].

To estimate the network, the ggmModSelect function and Spearman correlation were employed within the RStudio environment. This combination was chosen because it is considered more effective for estimating asymmetric data [80]. Next, centrality indices were examined. First, Expect Influence index (EI) was examined, which is preferred because the network contains negative signs and for such purposes is the most appropriate centrality index [81]. Second, in order to evaluate the nodes in different communities, we preferred to use the Bridge Expected Influence (BEI) index was preferred, which is the sum of edges (considering signs) between a node and other nodes outside its community [82]. Other centrality measures such as closeness and betweenness were not estimated due to their inadequacy for interpreting psychological variables [83] and their instability according to simulation studies [84]. It is important to note that the network is represented by nodes (circles) connected by edges (lines), with varying thickness to denote the strength of the interaction. Positive and negative correlations are denoted by green and red colors, respectively [85]. The Fruchterman-Reingold algorithm was used to arrange the nodes, in which stronger interactions are centralized and weaker ones are placed on the periphery [86]. R2 predictability indices were included in the estimations to indicate the percentage of variance explained by each node with other nodes in the network [26].

The evaluation of edge weight accuracy entailed the implementation of the bootstrapping technique, a rigorous statistical resampling method, facilitated by the bootnet package. This method entailed iteratively modeling data randomly selected from the dataset, with edge values estimated in each iteration. To ascertain the precision of the edges, confidence intervals (CIs) were computed at a 95% level, unveiling the width of the intervals as a reflection of the accuracy level [87]. Furthermore, a comprehensive visual representation, in the form of a plot, was devised to depict the frequency at which edges were unequivocally assigned a zero value.

The assessment of stability encompassed a meticulous analysis of a plot elucidating the fluctuations in centrality indices after the removal of a staggering 70% of the data. Subsequently, a meticulous comparative analysis ensued, contrasting the resampled data against the original study data through the computation of their correlation mean. This intricate process culminated in the derivation of a comprehensive summary statistic, encapsulated within the Stability Correlation (CS), serving as a paramount metric discerning the extent to which data can be excised whilst upholding a commendable correlation threshold of at least 0.70 with the centrality coefficients of the data. It is imperative to emphasize that the final CS value is anticipated to reside within the prescribed range of 0.25 ≤ CS ≤ 0.50, illuminating the robustness and reliability of the stability assessment [85].

A comparison was conducted according to gender, and since there is a significant difference between the groups, a statistical technique for unbalanced data was used. The preferred technique for synthetic oversampling was Synthetic Minority Over-sampling Technique (SMOTE) because it has demonstrated good performance with extremely imbalanced [88] and categorical or ordinal data [89]. In addition, we used the NetworkComparisonTest library package [90]. This package employs a permutation procedure to test the null hypothesis that both groups are identical, examining the differences after generating one thousand randomly obtained replicates. To determine effect size, Spearman correlations based on bootstrap were established, and the mean of the correlations obtained from one thousand resamples was reported. Furthermore, differences between the two networks were investigated by subtracting the values from the matrix, and visualized in a corPlot graph, which allowed for the immediate identification of the most significant differences.

## Results

### Global network properties

The density analysis of the studied network revealed that 13 out of the 21 edges had a non-zero value, resulting in a density of 61.90%. A transitivity coefficient of 0.62 was found, indicating a good proportion of closed triangles in the network, higher than the random transitivity of 0.53. Regarding the average shortest path length (APL), on average, 1.43 links are required to reach from one node to another, suggesting high efficiency in information transmission. Finally, the small-world index obtained was 1.25, indicating proximity between nodes and efficient information propagation in the network.

### Estimation of the network and centrality

**Fig. 1:**
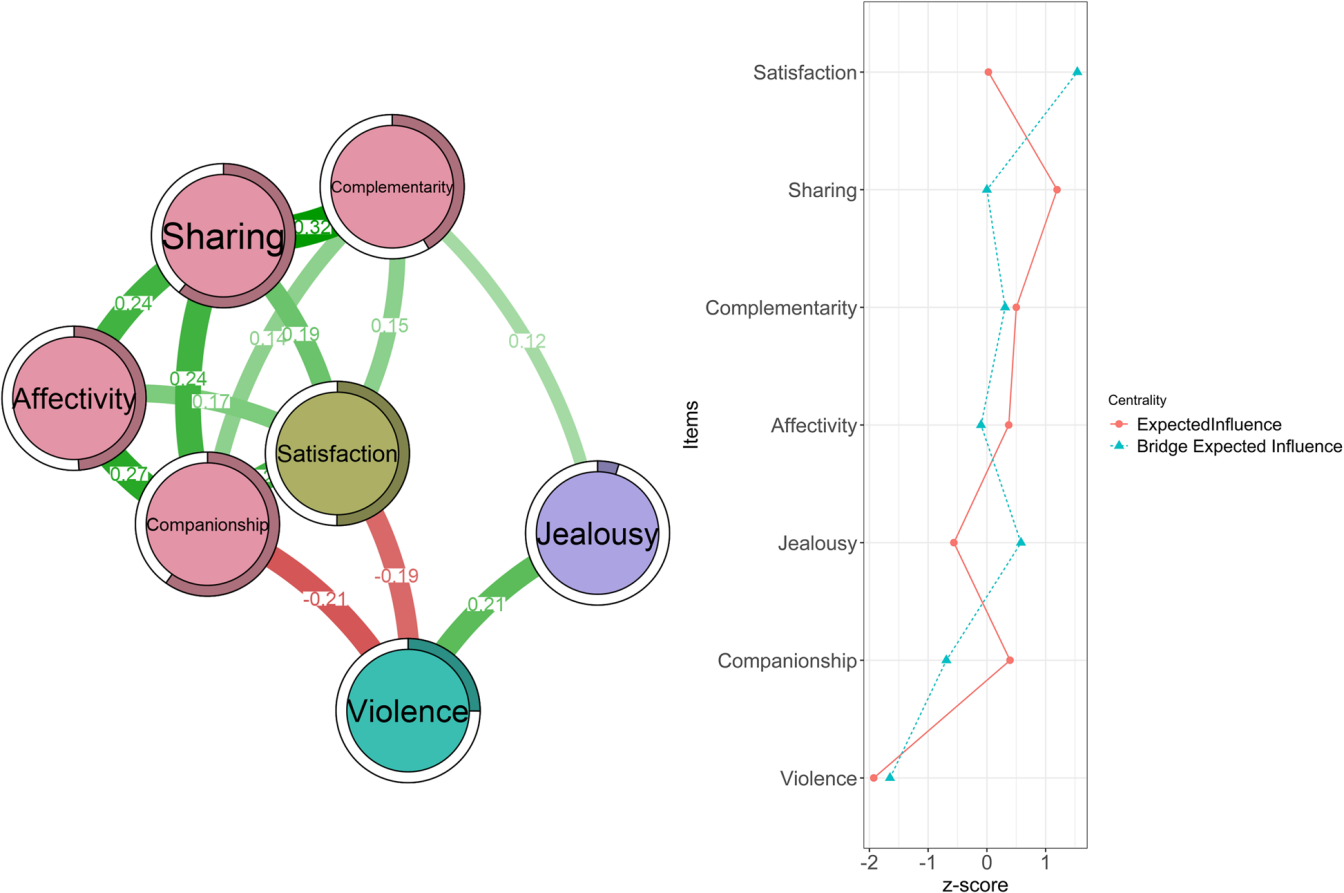
Network estimation and centrality indice. Note: The full name of Sharing is Shared interaction

In Fig. 1, it can be observed that the dimensions of relationship maintenance have mostly moderate relationships. The strongest relationship is found between Complementarity-Sharing (*r* = 0.32). On the other hand, smaller relationships are observed between Companionship-Affectivity (*r* = 0.27), Companionship-Sharing (*r* = 0.24), and Affectivity-Sharing (*r* = 0.24). The weakest relationship within these dimensions is found between Companionship-Complementarity (*r* = 0.14). In relation to other variables, both Complementarity-Jealousy (*r* = 0.12) and Companionship-Violence (*r* = -0.21) exhibit small relationships. Lastly, small relationships are also identified between Satisfaction-Violence (*r* = -0.19) and between Violence-Jealousy (*r* = 0.21). Regarding the central node, for the EI, the most central node is Sharing; while, for BEI Satisfaction, Satisfaction is identified as the central node according to Bridge Strength. These relationships align with the predictability measures obtained by R2, which are displayed as a bar on the edge of the node's circle.

### Stability and precision of the network

**Fig. 2:**
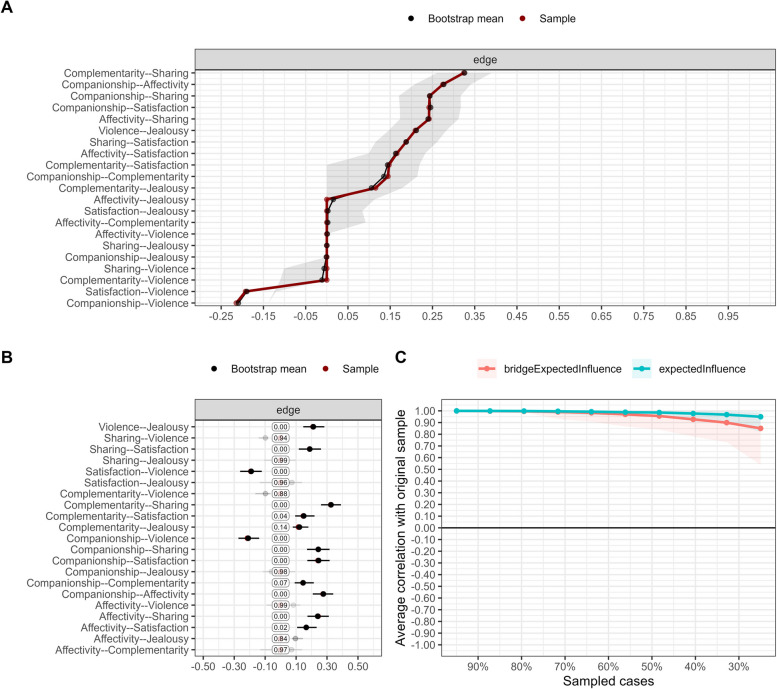
Network stability and accuracy

Figure 2A displays the accuracy of the edges, achieved by contrasting the mean relationship obtained from resampling (bootstrap mean) with the relationships derived from the original sample. Interestingly, a convergence of the black and red lines is observed, implying a plausible level of accuracy in the associations. Furthermore, the gray-colored band indicates a narrow confidence interval, suggesting minimal variation in the resampling process.

In Fig. 2B, a meticulously crafted graph illustrates the frequency at which parameters are set to zero and their corresponding frequencies. Surprisingly, the Affectivity-Complementarity link was nullified from the network in 97% of cases. The transparency of the interval denotes its infrequent inclusion, though when included, it was estimated to be of small but positive magnitude. On the contrary, the Violence-Jealousy connection exhibited robustness, never being eliminated from the network. The black shading of the interval signifies its constant presence in the network, with an estimation close to 0.20.

Figure 2C provides a visual representation showcasing the inherent stability of the centrality index obtained through resampling. Notably, there is a clear elevation above the 0.70 threshold in the average correlation between the original data and the data obtained through resampling, maintaining its high value even when cases are excluded. This holds true for both the EI and BEI indices. The stability coefficient (SC) reaches a value of 0.75 and 0.67 for EI and BEI, respectively, effortlessly surpassing the recommended minimum threshold of 0.50, further confirming the solidity and reliability of the analysis.

### Comparison

**Fig. 3:**
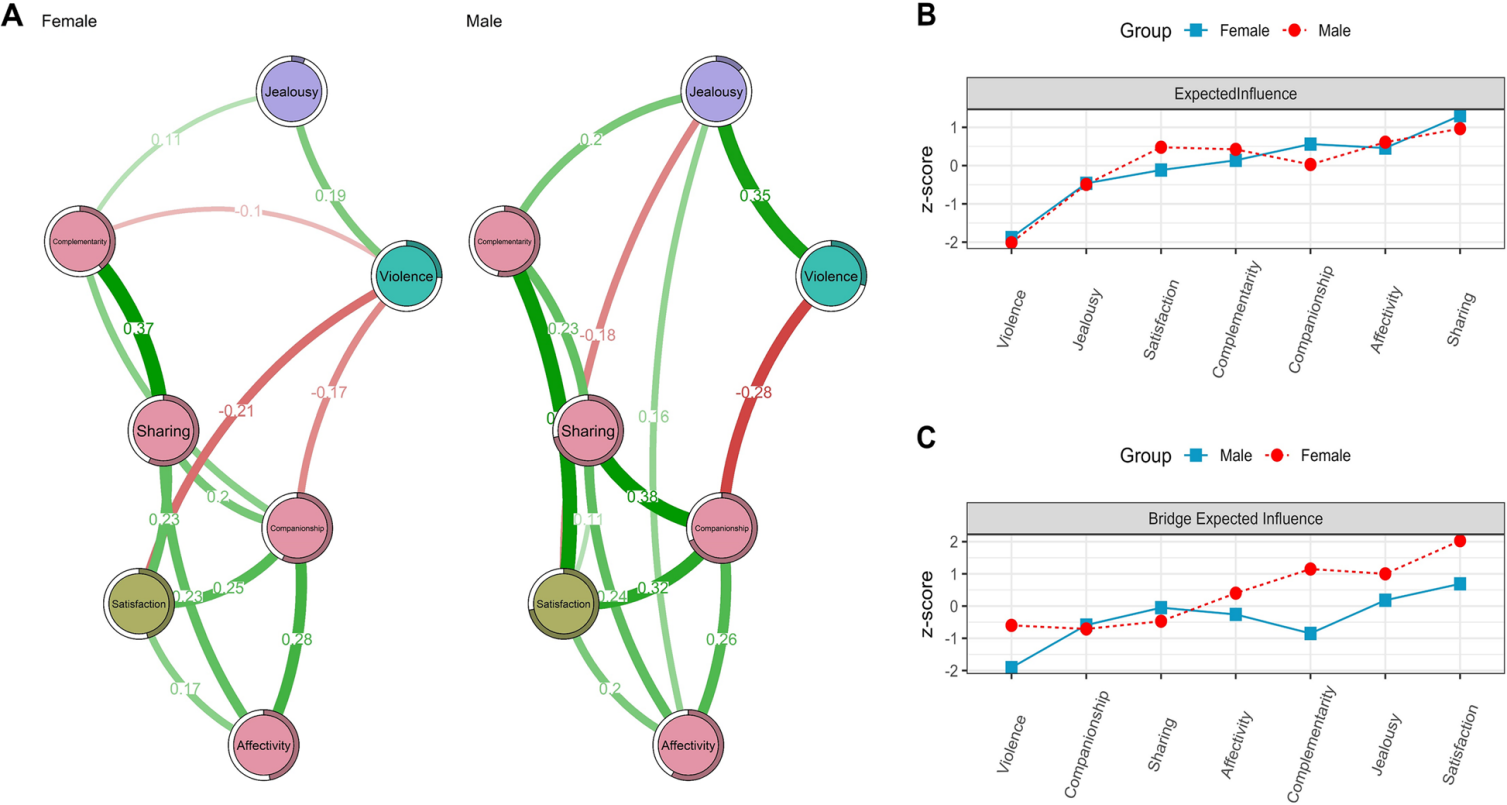
Networks according to gender

Figure 3 illustrates the stratified comparisons of the networks by gender. Notably, a discernible variation is observed in terms of statistical significance (M = 0.37; *p* < 0.001). In addition, connectivity is not identical (S = 0.60; *p* < 0.001). Interestingly, despite these disparities, the data matrices exhibit a significant similarity, as the average correlation derived from 1000 bootstrapped matrices for each network manifests a commendable value of 0.68.

When comparing men and women, differences in relational dynamics are evident. Men have a stronger link between Companionship and Sharing than women and a more pronounced association with Satisfaction and Violence. The interplay between Violence and Jealousy is also more distinct in men than women. Conversely, women have a unique connection between Companionship and Complementarity and a stronger bond between Sharing and Complementarity than men (see Fig. 3).

In relation to the centrality indexes, it is observed that the highest EI is Sharing and in the case of the EIB Satisfaction, both for men and women.

## Discussion

The present study examines the relationship between Relationship Maintenance, Satisfaction, Jealousy, and Violence in young Peruvian couples using a network analysis approach, which allows for the analysis of associations between nodes and facets [59, 85, 91]. Due to the lack of research in this field, the interaction between variables in romantic relationships is explored. Our study seeks to bridge this research gap, offering insights into the evolving dynamics of young couples' relationships in the aftermath of the pandemic. The resulting network exhibits an efficient and cohesive structure that facilitates the spread of information between nodes according to Global Network Properties.

The first objective was to identify the interconnections between each node in the network. It can be observed that two dimensions of Relationship Maintenance, namely Complementarity and Companionship, are associated with other variables such as Jealousy and Violence. The direct relationship between Complementarity and Jealousy underscores a significant shift in the dynamics of young couples' relationships post-pandemic. It hints at the possible emotional and social repercussions that such a global crisis may have induced, making these findings especially crucial in the broader context of relationship research. Therefore, the relationship between Complementarity and Jealousy suggests that intense sharing of interests and preferences can lead to unhealthy dependence and excessive desire for exclusivity in the relationship [92]. On the other hand, the inverse relationship between Companionship and Violence suggests that emotional and friendly connection in a relationship is contrary to episodes of aggression, tension, and difficulties in the romantic relationship. This is not surprising, as companionship is a fundamental characteristic of romantic relationships and refers to a longing for long-term commitment with another person [19]. Regarding the relationship between other variables, it is observed that Violence is related to Satisfaction and Jealousy. This is expected because abusive behaviors can negatively impact relationships [26]. In fact, jealousy and distrust are forms of negative relationship maintenance [29, 30]. Consequently, in the presence of violence, relationships are less likely to have satisfactory levels of trust and closeness, hindering the full development of the couple as it reflects coercive methods and hampers the satisfaction of both individual and relational needs [36]. Given that jealousy is closely tied to anger, relationships are likely to turn into destructive behaviors towards partners, leading to aggression in the relationship [93].

The second objective was to identify the central node and the bridge node in the network. In this regard, it was found that Sharing is the central node in the network and Satisfaction is the bridge node. Firstly, sharing is considered by some authors as one of the five behaviors for the success of romantic relationships [13], and it is known that women lean slightly more towards seeking companionship and sharing experiences and conversations [41]. Moreover, having quality time benefits the couple's relationship and is fundamental for individual enjoyment, associating with individual happiness [42, 43]. On the other hand, the fact that satisfaction is considered a bridging node is supported by previous studies that establish a relationship between relationship satisfaction and maintenance behaviors [20]. Given the importance of satisfaction in predicting the durability and success of romantic relationships [22], as well as its relevance alongside love as essential aspects in romantic relationships [23], it is interesting to continue investigating the centrality of satisfaction in relationships. Especially following the end of the pandemic, during which dating and married couples experienced a significant decrease in their level of satisfaction [24], and, as a result, a significant increase in conflicts was observed [94] and higher divorce rates [95]. These findings reinforce the idea that the pandemic has negatively impacted couple relationships [96]. In fact, according to a study comparing marriage records in 2020 with those of 2019, a dramatic decline in marriages was found in the first year of the pandemic [97]. Now, the effects of lockdowns, social distancing, and the resultant emotional and financial pressures have added a new dimension to this paradigm. Our study aids in comprehending these shifts and their implications on young couples.

A third objective was to compare the nodes by gender. As a result, it was found that the networks are not invariant, and the adjacency matrices are not similar. The study yielded interesting findings on gender. Men emphasize more the bond between Companionship and Sharing, perhaps because, culturally, they do not tend to share emotions as openly as women, nor seek support from others [98, 99]. Thus, they see the act of sharing and companionship as something relevant that does not occur in any other context of their lives. This data is novel and needs more research to be conclusive. Men also show strong links between Satisfaction, Violence and Jealousy, coinciding with studies linking male jealousy with anger or aggression [31, 32]. Women, on the other hand, have an outstanding relationship between Companionship and Complementarity, perhaps reflecting a valuing of similar interests and mutual support, given that they tend to feel more dissatisfaction and initiate divorces more quickly [45, 100]. This could explain why the link between Sharing and Complementarity is stronger in women.

The findings of this research have important theoretical and practical implications. The fact that Satisfaction is the bridge node aligns with models that emphasize the role of this variable in the durability and success of romantic relationships [22]. These findings are particularly interesting to examine as both dating and married couples experienced a significant decrease in their levels of satisfaction and love following the conclusion of the COVID-19 emergency [24]. From a practical point of view, these results highlight the importance of maintenance variables such as Companionship, Complementarity and Sharing. Especially the latter, which ended up being a central node in the whole network and involves spending time with friends and family, as well as sharing disagreements or events [66]. In fact, the absence of shared interaction may be a risk factor for satisfaction, violence reducing the likelihood of trust and closeness [36] and jealousy maintains the relationship but in a negative way [29, 30]. Drawing from our research, we unearth pivotal insights that can guide the development of interventions or strategies for young couples in the post-COVID-19 era. By shedding light on the pronounced links between elements such as complementarity and heightened jealousy, or the association between companionship and decreased tendencies for violence, experts can devise tailored counseling approaches or therapeutic solutions, addressing the unique challenges young couples confront following the pandemic's wake.

It is essential to keep in mind certain limitations in the findings of this research. Initially, participants were chosen using a non-probability snowball sampling design, as it was the only feasible alternative during the study period. Such designs have been frequently adopted in the domain of psychology [101]. Furthermore, implementing random sampling is challenging in a virtual environment. Secondly, the disparity in the sample between men and women is a result of the sampling design, without controlling for proportional representation in the sample. Although, we have used an algorithm for unbalanced data such as SMOTE, it is suggested that future research should strive for gender equality to examine the stability of the results found here.

In conclusion, this study has undertaken a comprehensive examination of the interplay between relationship maintenance, satisfaction, jealousy, and violence within a cohort of young Peruvian couples, utilizing a network analysis paradigm. The resulting network unveils an efficient and highly organized structure, fostering information dissemination across the nodes. Importantly, the findings reveal interconnections between diverse variables, exemplified by the direct link between complementarity and jealousy, as well as the inverse association between companionship and violence. Notably, satisfaction emerges as the prominent and fundamental central node within the network, aligning with its well-established significance in the realm of romantic relationships. Additionally, the study shows men prioritize Companionship and Sharing, possibly due to cultural norms. They also link Satisfaction with Violence and Jealousy, while women focus on the Companionship-Complementarity bond, indicating mutual support. These findings carry profound theoretical and practical implications, underscoring the importance of maintenance variables and the imperative to explore both positive and negative dynamics within the intricate domain of romantic relationships. These insights assume heightened relevance in the current pandemic context, which has undoubtedly engendered profound repercussions on human relationships.

### Supplementary Information

**Additional file 1. **WAST analysis.

## Data Availability

The datasets generated during and/or analyzed during the current study are available from the corresponding author on reasonable request.
