# Supplementary material for: Maintenance in relationships, satisfaction, jealousy, and violence in young couples: a network analysis
Source: BMC Psychol. 2023 Nov 9;11:385. doi: 10.1186/s40359-023-01411-z (PMC10634140; doi:10.1186/s40359-023-01411-z)

WAST Analysis

anonimo

2023-10-20

libraries

library(tidyverse)
library(mirt)

Oversampling to match male to female data

df <- readxl::read_excel("Maintenance_data.xlsx")
data <- df %>% dplyr::select(WAST1, WAST2, Sex) %>% as.data.frame()
outcome <- "Sex" # The column you want to predicts
set.seed(2023)
newdata <- RSBID::SMOTE(data, outcome, perc_maj = 100, k = 3)
newdata %>% count(Sex)

## Sex n
## 1 Mujer 645
## 2 Varon 645

Model estimation

# selection of number of males and femaless
set.seed(4)
group <- newdata %>% dplyr::select(WAST1:WAST2, Sex) %>%
 group_by(Sex) %>% pull(Sex) #Reference group = Women; Focus group = Males
#Calculate a multigroup model
model_group <-multipleGroup(newdata %>% dplyr::select(WAST1:WAST2),
 model = 1,
 group = group,
 SE = TRUE,
 itemtype = "graded",
 verbose = FALSE,
 type = "G2")
model_group

##
## Call:
## multipleGroup(data = newdata %>% dplyr::select(WAST1:WAST2),
## model = 1, group = group, itemtype = "graded", SE = TRUE,
## verbose = FALSE, type = "G2")
##
## Full-information item factor analysis with 1 factor(s).
## FAILED TO CONVERGE within 1e-04 tolerance after 500 EM iterations.
## mirt version: 1.38.1
## M-step optimizer: BFGS
## EM acceleration: Ramsay
## Number of rectangular quadrature: 61
## Latent density type: Gaussian
##
## Information matrix estimated with method: Oakes
## Second-order test: model is a possible local maximum
## Condition number of information matrix = 7041.72
##
## Log-likelihood = -2104.755
## Estimated parameters: 12
## AIC = 4233.51
## BIC = 4295.459; SABIC = 4257.341
## G2 (4) = 15.48, p = 0.0038
## RMSEA = 0.047, CFI = NaN, TLI = NaN

DIF based on Differential Response Functioning statistics.

#DIF por DRF
DRF_model <- DRF(model_group, draws = 1000, DIF=T, plot=F, theta_lim = c(-6,6)) #DIF=T por item
DRF_model

Extraction of information from the DRF

#Extract information
A <- bind_cols(
 DRF_model$uDIF %>% as_tibble() %>% dplyr::select(uDIF),
 DRF_model$sDIF %>% as_tibble() %>% dplyr::select(sDIF)
)
# %>% openxlsx::write.xlsx(., file = "DRF.xlsx", overwrite = T)
B <- mirt::empirical_ES(model_group) %>% dplyr::select(ESSD)

bind_cols(A,B)

## # A tibble: 2 x 3
## uDIF sDIF ESSD
## * <dbl> <dbl> <dbl>
## 1 0.170 0.0584 0.147
## 2 0.0608 0.0608 0.115

Effect size

#Para el test
mirt::empirical_ES(model_group, DIF = F) %>% dplyr::filter(`Effect Size` == "ETSSD")

## Effect Size Value
## 1 ETSSD 0.131676

#Para los items
mirt::empirical_ES(model_group, DIF = T)

## SIDS UIDS SIDN UIDN ESSD theta.of.max.D max.D mean.ES.foc
## item.1 0.062 0.188 0.058 0.169 0.147 1.825 0.571 0.800
## item.2 0.051 0.051 0.062 0.062 0.115 1.324 0.177 0.823
## mean.ES.ref
## item.1 0.738
## item.2 0.772

#plots
plot(model_group)


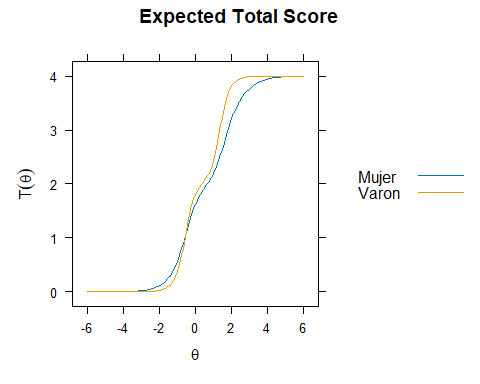


plot(model_group, type = 'itemscore', which.items = 1:2)


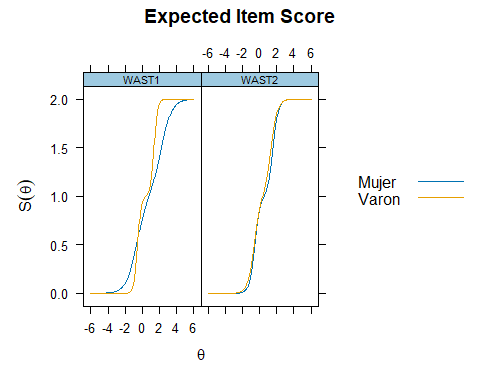

Supplement: Supplementary file 1 — Additional file 1. WAST analysis. [file 40359_2023_1411_MOESM1_ESM.docx]
